# RETRACTION: Comparison of the Incidence of Wound Complications With Preoperative and Postoperative Radiotherapy in Patients With Extremity Soft Tissue Sarcoma Resection: A Meta‐Analysis

**DOI:** 10.1111/iwj.70326

**Published:** 2025-03-06

## Abstract

**RETRACTION:** YanH.‐K. , HuangJ. , YangZ.‐H. , ChenW.‐G. , XiaZ.‐D. , XiangY. , and PengH. , “Comparison of the Incidence of Wound Complications With Preoperative and Postoperative Radiotherapy in Patients With Extremity Soft Tissue Sarcoma Resection: A Meta‐Analysis,” International Wound Journal 21, no. 2 (2024): e14441, 10.1111/iwj.14441.PMC1082812737853943

The above article, published online on 19 October 2023, in Wiley Online Library (http://onlinelibrary.wiley.com/), has been retracted by agreement between the journal Editor in Chief, Professor Keith Harding; and John Wiley & Sons Ltd. Following an investigation by the publisher, all parties have concluded that this article was accepted solely on the basis of a compromised peer review process. The editors have therefore decided to retract the article. The authors did not respond to our notice regarding the retraction.

**RETRACTION:** H.‐K. Yan , J. Huang , Z.‐H. Yang , W.‐G. Chen , Z.‐D. Xia , Y. Xiang , and H. Peng , “Comparison of the Incidence of Wound Complications With Preoperative and Postoperative Radiotherapy in Patients With Extremity Soft Tissue Sarcoma Resection: A Meta‐Analysis,” International Wound Journal 21, no. 2 (2024): e14441, 10.1111/iwj.14441.PMC1082812737853943

The above article, published online on 19 October 2023, in Wiley Online Library (http://onlinelibrary.wiley.com/), has been retracted by agreement between the journal Editor in Chief, Professor Keith Harding; and John Wiley & Sons Ltd. Following an investigation by the publisher, all parties have concluded that this article was accepted solely on the basis of a compromised peer review process. The editors have therefore decided to retract the article. The authors did not respond to our notice regarding the retraction.

